# Novel Mixed-Dimensional hBN-Passivated Silicon Nanowire Reconfigurable Field Effect Transistors: Fabrication and Characterization

**DOI:** 10.1021/acsami.3c04808

**Published:** 2023-08-22

**Authors:** Sayantan Ghosh, Muhammad Bilal Khan, Phanish Chava, Kenji Watanabe, Takashi Taniguchi, Slawomir Prucnal, René Hübner, Thomas Mikolajick, Artur Erbe, Yordan M. Georgiev

**Affiliations:** † Institute of Ion Beam Physics and Materials Research, Helmholtz-Zentrum Dresden-Rossendorf (HZDR), Bautzner Landstraße 400, Dresden 01328, Germany; ‡ Technische Universität Dresden, Dresden 01069, Germany; § National Institute for Materials Science, 1-1 Namiki, Tsukuba 305-0044, Japan; ∥ Namlab gGmbH, Nöthnitzer Strasse 64, Dresden 01187, Germany; ⊥ Technische Universität Dresden, Center for Advancing Electronics Dresden (CfAED), Dresden 01069, Germany; # Institute of Electronics at the Bulgarian Academy of Sciences, 72 Tsarigradsko chaussee blvd, Sofia 1784, Bulgaria

**Keywords:** mixed-dimensional reconfigurable FET, ambipolar, nickel silicide, flash lamp annealing, hBN encapsulation, subthreshold swing

## Abstract

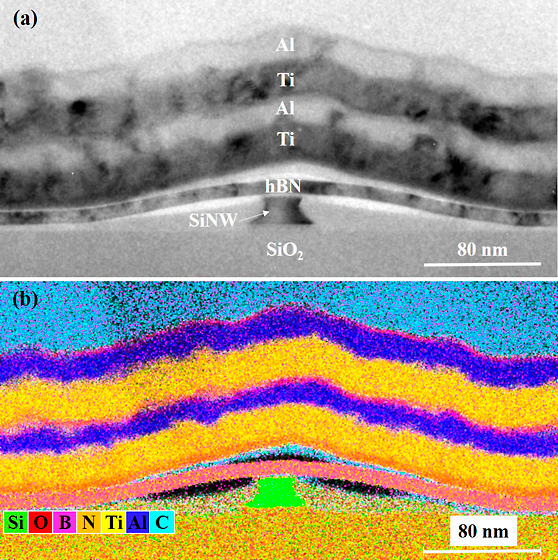

This work demonstrates the novel concept of a mixed-dimensional reconfigurable field effect transistor (RFET) by combining a one-dimensional (1D) channel material such as a silicon (Si) nanowire with a two-dimensional (2D) material as a gate dielectric. An RFET is an innovative device that can be dynamically programmed to perform as either an n- or p-FET by applying appropriate gate potentials. In this work, an insulating 2D material, hexagonal boron nitride (hBN), is introduced as a gate dielectric and encapsulation layer around the nanowire in place of a thermally grown or atomic-layer-deposited oxide. hBN flake was mechanically exfoliated and transferred onto a silicon nanowire-based RFET device using the dry viscoelastic stamping transfer technique. The thickness of the hBN flakes was investigated by atomic force microscopy and transmission electron microscopy. The ambipolar transfer characteristics of the Si-hBN RFETs with different gating architectures showed a significant improvement in the device’s electrical parameters due to the encapsulation and passivation of the nanowire with the hBN flake. Both n- and p-type characteristics measured through the top gate exhibited a reduction of hysteresis by 10–20 V and an increase in the on–off ratio (*I*_ON_/*I*_OFF_) by 1 order of magnitude (up to 10^8^) compared to the values measured for unpassivated nanowire. Specifically, the hBN encapsulation provided improved electrostatic top gate coupling, which is reflected in the enhanced subthreshold swing values of the devices. For a single nanowire, an improvement up to 0.97 and 0.5 V/dec in the n- and p-conduction, respectively, is observed. Due to their dynamic switching and polarity control, RFETs boast great potential in reducing the device count, lowering power consumption, and playing a crucial role in advanced electronic circuitry. The concept of mixed-dimensional RFET could further strengthen its functionality, opening up new pathways for future electronics.

## Introduction

The physical downscaling of silicon-based MOSFET technology has reached its limitations. Subsequently, the quest for alternative technological solutions based on new materials and device concepts augments the downscaling of integrated circuits. One such state-of-the-art advancement that has been intensively researched in the past decade to improve the functionality of nanowire field effect transistors (FETs) is called reconfigurability.^1^ Reconfigurability is based on electrostatic control of the transistor’s polarity. Transistors that are built on this concept are called reconfigurable FETs (RFETs).^2^ A nanowire RFET is a Schottky junction-based device that can be reversibly configured to n- or p-polarity by controlling the electrostatic potential applied at the polarity gates. Due to this property, the functional complexity of a system can be enhanced by these transistors without increasing the device count. In the most generic process, the device is based on an intrinsic Si nanowire with nickel (Ni) contact pads placed on both ends. Subsequent annealing results in the formation of silicide regions in the nanowire. Consequently, silicide-Si-silicide Schottky junctions are formed. By control of the Schottky barrier at the two ends of the nanowire with the help of gate potentials, the type and flow of charge carriers within the channel can be dynamically modulated. For ambipolarity, an electrostatic potential on the back gate or a single top gate enables the n- or p-transport, depending on the polarity of the gate voltage.

**Figure 1:**
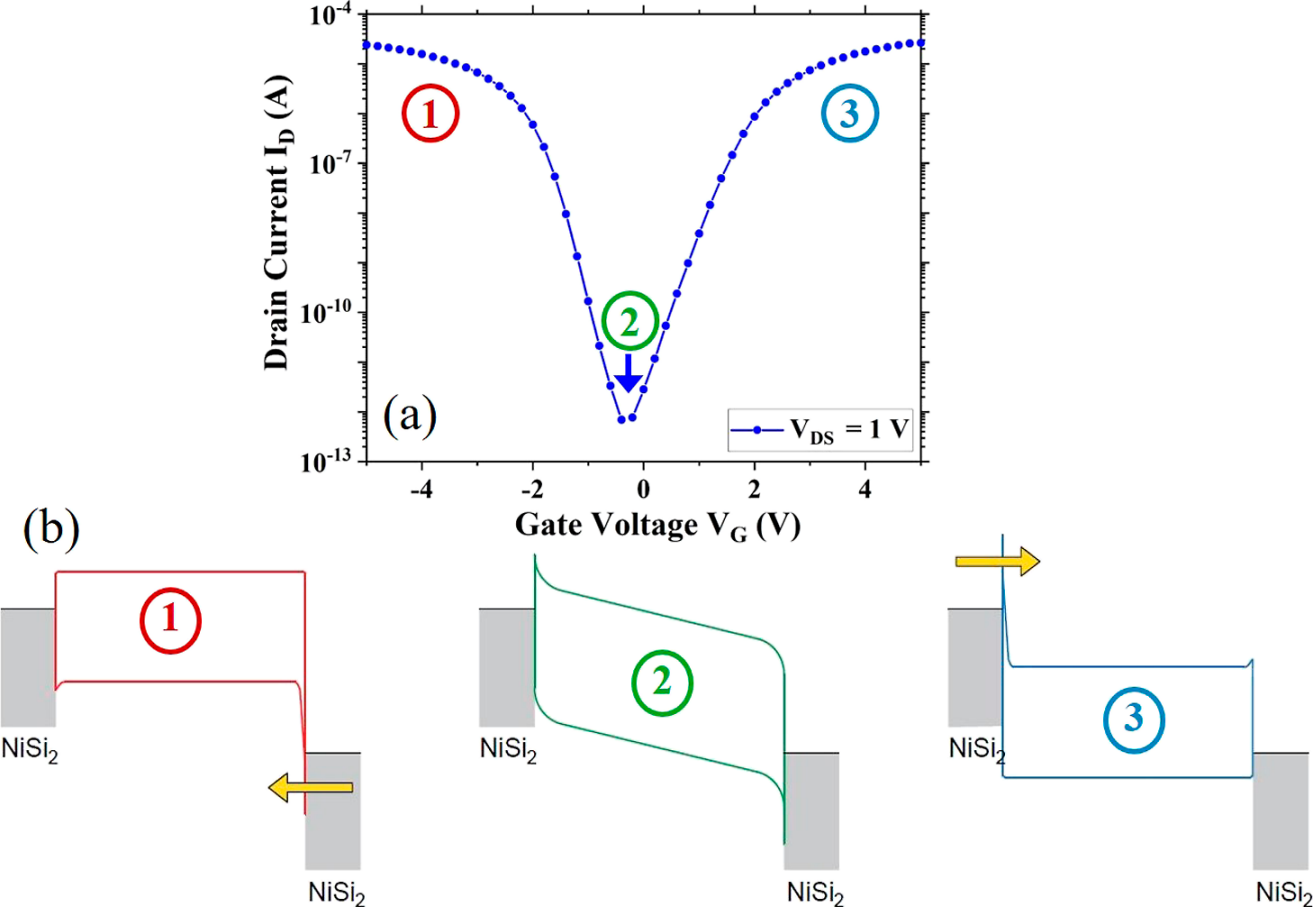
Ambipolar RFET characteristics (a) typical IV characteristics of a silicon nanowire RFET (b) energy band diagram of an ambipolar RFET under biased condition showing (1) hole conduction by tunneling with upward band bending, (2) off-state region dominated by high energy charge carrier conduction, and (3) electron conduction by tunneling with downward band bending.

An ambipolar RFET device can be switched to its ON state by the application of both positive and negative voltages. This nature of switching on the RFET device by sweeping the gate voltage from a high potential to a low potential is known as ambipolarity.^3^ In ambipolar devices, both types of charge carriers take part in the conduction in a single voltage sweep. The ambipolar RFET realized in this work is predominantly single-gated. This gate can be either a common back gate or a fabricated top gate. The gate electrode covers the Schottky junction region on either side of the nanowire channel. By applying a suitable voltage on the gate, the Schottky barrier is tuned accordingly to steer charge carriers into the channel for current conduction. In the OFF state of the device, the presence of both electron and hole barriers causes a limited off-current to flow, which is mainly due to high energy thermionic emission. When a positive bias voltage is applied to the gate structure, the energy of the bands is lowered, reducing the barrier height for the electrons. This leads to the injection of electrons into the channel by tunneling, contributing to an electron current or n-type conduction taking place. Simultaneously by sweeping the gate bias voltage to a low negative potential, the energy bands rise, reducing the barrier height for holes and promoting hole conduction. As the conduction band edge also rises at the same time, the electrons are blocked due to the high electron barrier height. Hence, the current is totally dominated by hole conduction only and is termed p-type conduction. The IV characteristics of an ambipolar RFET device and the band bending at different bias voltages are depicted in Figure 1.

As a single-gate architecture is used for ambipolar operation, the preferred gate structure is mostly the top gate. The top gate provides a better field effect capacitive coupling over the Schottky junctions and the nanowire channel than the back-gated architecture. This is due to the difference in thickness of the top gate dielectric and the buried oxide layer. The relation between the capacitance and the thickness of the dielectric is given by the following equation
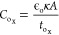
1where  is the oxide capacitance which corresponds to capacitive coupling of the gate over the channel, ϵ_o_ represents the relative permittivity of free space, κ is the dielectric constant, *A* is the area, and  represents the thickness of the oxide or dielectric. Due to the lower thickness value of the top gate dielectric, a higher capacitive control of the channel is achieved, requiring a much smaller operational voltage for switching the device on. This also improves the subthreshold swing (SS) of the device.

Contrary to conventional transistors, charge carrier injection in the channel is selectively controlled at the Schottky junctions, thereby avoiding the need of having an inherently doped channel material. Tuning the Schottky barrier at both ends of the nanowire is one of the basic requirements for current transport in reconfigurable devices. This is achieved by electrostatic coupling and enhanced gate control over the channel material with a proper encapsulating and passivating dielectric material. Such transistors have been demonstrated using different channel materials such as carbon nanotubes,^4^ graphene,^5^ silicon (either as nanowires, utilizing FinFET or fully depleted silicon-on-insulator (FDSOI) transistors to achieve a high gate coupling),^6–9^ and 2D semiconductors.^10^ However, exploration of dielectric materials is relatively scarce.

The scientific exploration of 2D materials after the isolation of graphene in 2004^11^ paved the way for a promising option in the field of nanoelectronics due to their stability at atomic thickness, high intrinsic mobility, optical transparency, and high strain limit compared to conventional semiconductors and insulators. One such material, which has excellent insulating properties with a large direct band gap of about 6 eV, is hexagonal boron nitride (hBN).^12,13^ 2D hBN has a structure analogous to graphene much like a honeycomb maze and is therefore known as “white graphene.” The hexagonal sublattice of hBN consists of alternating boron and nitrogen atoms with the electron-deficient boron atoms laying directly above the electron-rich nitrogen atoms or vice versa in adjacent layers.^14^ The asymmetry in the sublattices causes polarized covalent bonds leading to the large band gap of hBN, making it act as an insulator. Interlayer van der Waals forces dictate the thickness of multilayer hBN, and the mechanically exfoliated monolayer is about 0.4 nm thick.^14^ Atomically thin hBN has flat interfaces due to the absence of surface dangling bonds and is, therefore, resistant to oxidation. It also acts as a good gate insulator with a dielectric constant ranging between 3 and 4 (similar or comparable to that of SiO_2_)^15^ and can be even incorporated as an encapsulating layer for the active channel material of devices to prevent degradation of relatively unstable channel materials.^16^ hBN is chemically stable and can withstand 1000 °C at ambient conditions, 1400 °C in vacuum, and up to 2850 °C in an inert atmosphere.^14^ The thermal conductivity is about 484 W m^–1^ K^–1^ for 2D hBN with an elastic constant of 220–510 N m^–1^ and a Young’s modulus of about 1 TPa, thus making it an excellent material for flexible insulation.^17^

The top-down fabricated reconfigurable devices reported in this work are the first of their kind to incorporate silicon nanowires as the channel material along with 2D hBN as the gate dielectric and encapsulating layer. The focus of this work is on the development of the fabrication process, including structural studies of the device. Furthermore, the electrical transfer characteristics of such Si-hBN devices are measured based on different gating architectures to show a significant improvement in SS values, reduction of hysteresis, and increase in p and n on-currents due to the 2D encapsulation and passivation.

## Results and Discussion

**Figure 2:**
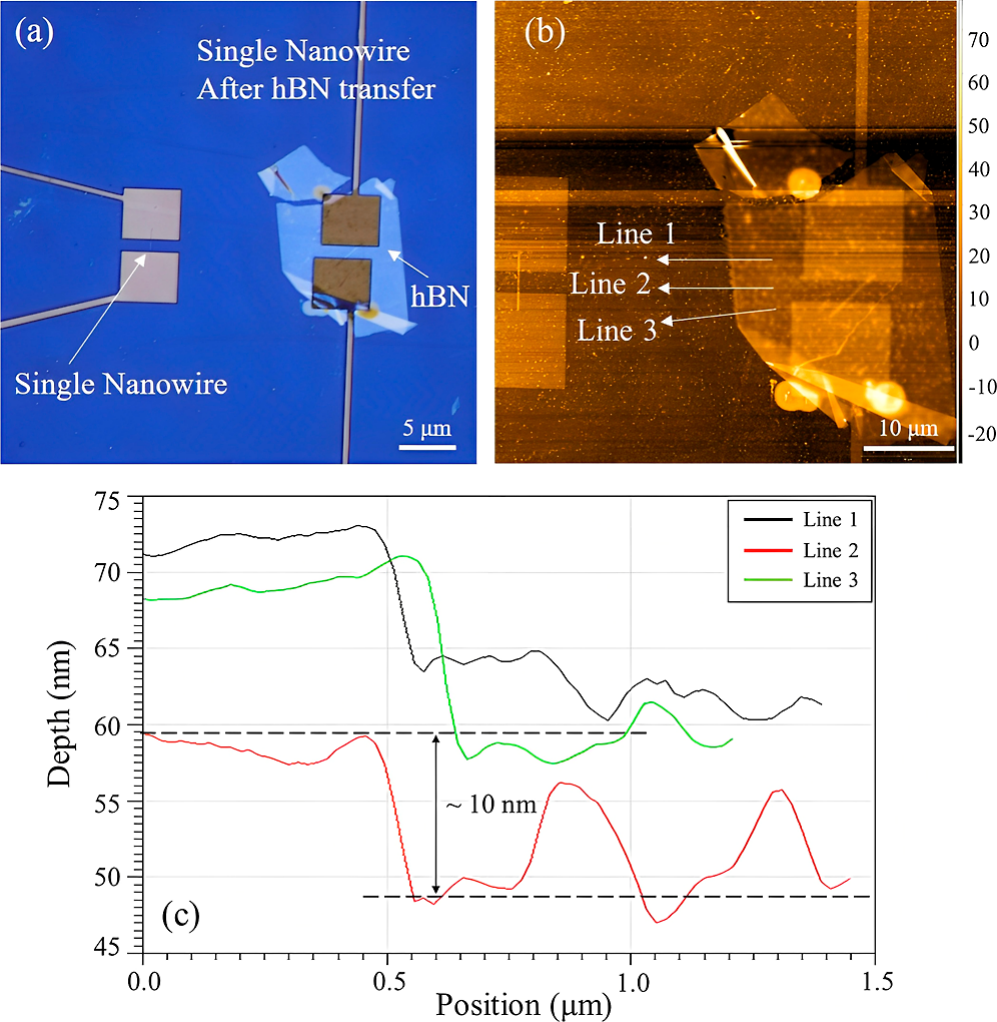
AFM characterization of the fabricated devices. (a) Visible light micrograph of two different single silicon nanowire devices with and without hBN dielectric layer. (b) AFM scan of the fabricated devices is shown in image (a). Lines 1, 2, and 3 are three different line profiles for determining the thickness of the hBN flake. (c) Thickness of hBN is based on the height profile lines 1, 2, and 3 in the image (b). Thickness of the hBN flake shown here is approximately 10 nm.

The initial structural characterization of the mixed-dimensional RFET devices includes tapping-mode atomic force microscopy (AFM) analysis. Height profiles are extracted from multiple AFM scans to determine the thickness of the hBN flakes. Figure 2a shows an optical micrograph of two single nanowire-based devices.^18^ The first device is without an hBN dielectric layer, while the second device is capped with a thin hBN layer. AFM analysis is performed on these two devices, and the AFM topography is shown in Figure 2b. Three individual line profiles are drawn from the edges of the hBN flake to determine its thickness. The thickness of the hBN flake is approximately 10 nm, as can be seen from Figure 2c.

**Figure 3:**
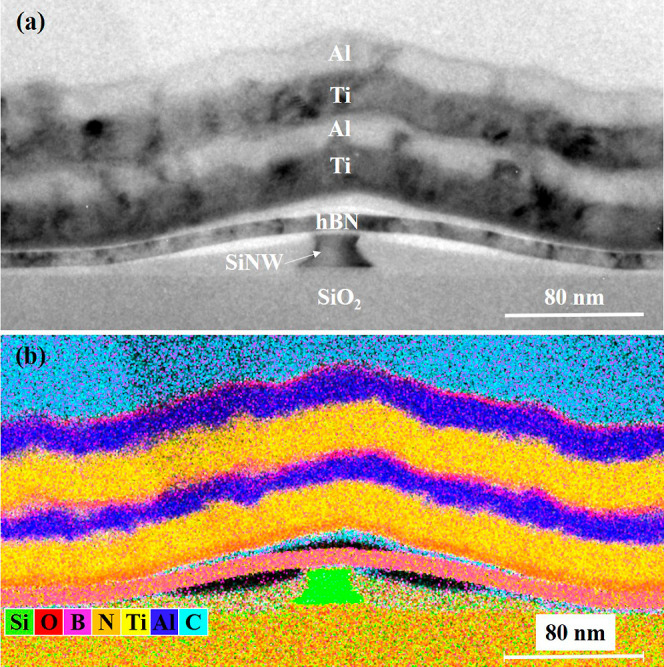
(a) Bright-field TEM micrograph of a sectioned nanowire device. The device structure consists of a buried SiO_2_ layer underneath the silicon nanowire. On top of the nanowire channel is the hBN dielectric, which is shown to cover the nanowire. A stack of Ti and Al serves as the gate electrodes above the hBN. (b) Corresponding superimposed EDXS-based element distribution maps of the hBN-silicon-nanowire-based device.

For further analysis of the hBN encapsulation of the nanowire, cross-sectional TEM analysis is performed. Figure 3 shows a representative bright-field TEM image. It is to be noted that the sectioning of the device is carried out across the single nanowire structure (i.e., perpendicular to the length of the nanowire). The cross-sectioning is performed to study and analyze the RFET device, especially the conformity of the hBN flake around the single nanowire. As seen from Figure 3a, the hierarchy of the RFET device starts with the buried SiO_2_ layer at the bottom of the single nanowire channel. The nanowire channel has a trapezoidal shape with a height and a width of about 20 and 25 nm, respectively. The hBN thickness is confirmed to be approximately 10 nm and is shown to cover the nanowire channel from the top. On top of the dielectric layer, a stack of titanium (Ti) and aluminum (Al) acts as the gate electrode for the device.

The bright-field TEM image shows the stretch of the hBN flake and the position where it finally sits on the buried oxide layer (away from the silicon nanowire channel). The structure of the top gate follows the curvature of the hBN flake. To study the element distribution in the sectioned device, a spectrum imaging analysis based on energy-dispersive X-ray spectroscopy (EDXS) is applied. The superposition of the corresponding element distribution maps of the device is shown in Figure 3b. The region enriched with boron (B) and nitrogen (N) confirms the presence of the hBN dielectric layer above the silicon nanowire channel. It is seen that the hBN flake sits on top of the silicon nanowire channel but does not conformally encapsulate its sidewalls. The EDXS-based analysis also confirms the presence of voids (denoted by the black areas in the element map). It is also to be noted that a dual layer of Ti and Al is evaporated on top of the hBN dielectric. The reason for using a dual-layer structure is that the first gate stack of Ti and Al was slightly misaligned with the silicon nanowire during the top gate fabrication. Hence, another broader layer of Ti and Al was precisely aligned and deposited above it for better capacitive coupling of the gate to the hBN stack. The growth of the oxide layer around the nanowire and in between the metal top gates (as seen in Figure 3b) is a likely phenomenon in ambient conditions. Such growth of oxide layers has also been outlined in previous studies regarding nanowire devices and silicidation.^19,20^ The presence of carbon (C) on top of the Ti and Al gate stack is due to the protective capping layer deposited during TEM specimen preparation. The formation of carbon with traces of silicon (Si) and oxygen (O) around the cross-section of the nanowire channel and above the hBN flake is mostly caused by the fabrication process of the device. Several scanning electron microscopy images were taken of the nanowire devices before and after hBN transfer in order to characterize the device. Thereby, organic substances on the surface of the sample were cracked and deposited as carbon layers.

**Figure 4:**
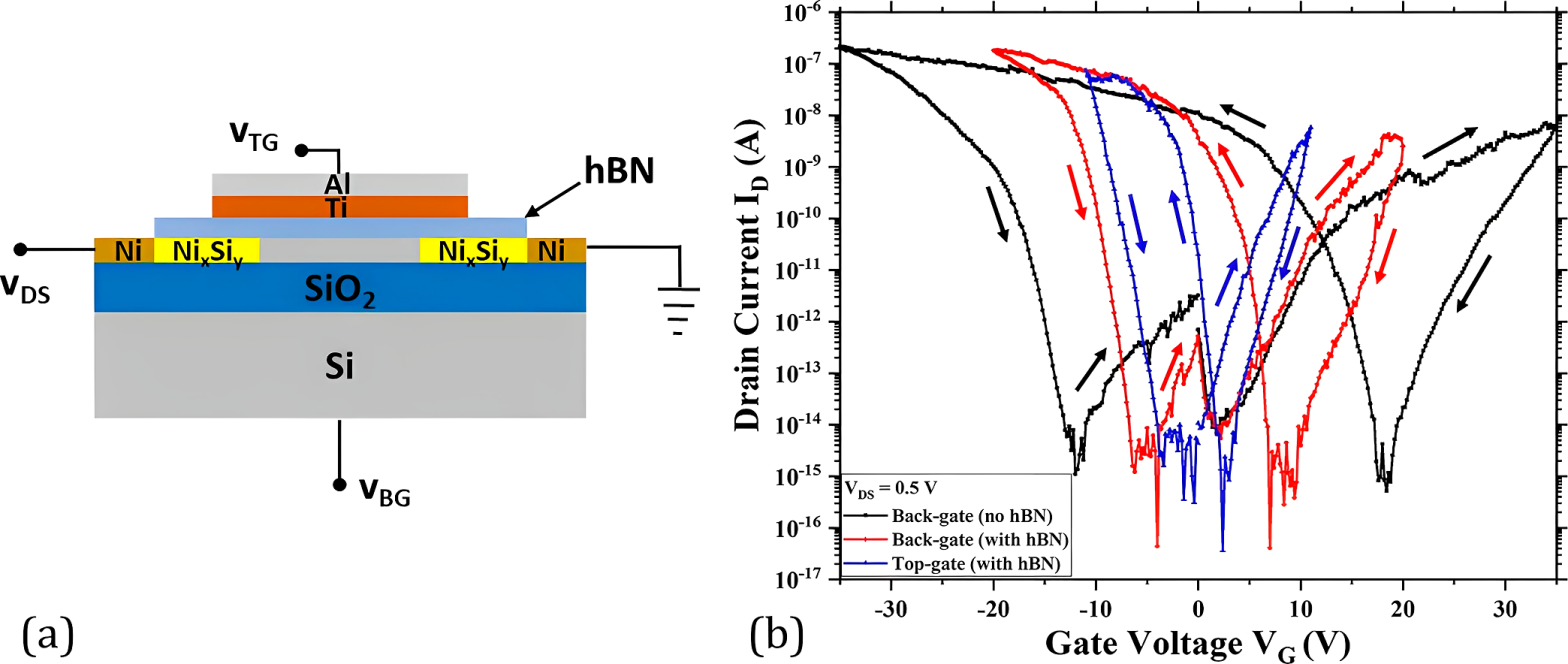
(a) Cross-sectional view of the device, including the voltage naming conventions used in this work. The device structure consists of a bottom Si layer on top of which is a 100 nm buried SiO_2_ layer. The single nanowire is fabricated on top of the buried oxide layer. Ni contact pads are placed on both sides of the Si nanowire. Annealing creates Ni_*x*_Si_*y*_ Schottky junctions in the nanowire. hBN is transferred on top of the device as the gate dielectric. A bilayer of Ti and Al serves as the top gate for the device. (b) Transfer characteristics of a single silicon nanowire-based device comprising an hBN flake as the dielectric layer. Different gating schemes are incorporated to deduce the IV characteristics. The length and width of the nanowire are 3 μm and 25 nm, respectively. The arrows in the transfer characteristics denote the direction of the curve based on the gate voltage sweep.

Furthermore, the effect of hBN as a dielectric layer for silicon nanowire-based devices on their electrical properties is investigated. For this, the electrical measurements are carried out in an ambient atmosphere. The measurement schematic and transfer characteristics of a single nanowire-based device are presented in Figure 4. Three types of transfer characteristics are obtained by the following measurement schemes:

1. Back gating the device before hBN is transferred. The back gate voltage (*V*_BG_) is swept between 35 and −35 V in a closed loop.
2. Back gating the device after the transfer of the hBN flake. *V*_BG_ is swept between 20 and −20 V in a closed loop.
3. Top gating the devices using hBN as the gate dielectric. The top gate voltage (*V*_TG_) is swept between 10 and −10 V in a closed loop.

Lower gate voltages (*V*_G_) are applied after hBN transfer to avoid the dielectric breakdown of the hBN. A drain to source voltage (*V*_DS_) of 0.5 V is used to obtain the transfer characteristics. The device showed ambipolar behavior with improved electrostatic gate coupling after hBN transfer, which results in an enhanced SS value of the device (Table 1). The best values of SS are obtained by top gating the device. It is also evident from Figure 4b that the shift of the minima of the transfer curves is clearly reduced (toward the origin (0 V)) after the transfer of the hBN flake. The characteristics also show an improvement in hysteresis after hBN transfer with the lowest hysteresis for the top-gated configuration. These are mainly due to the hBN passivation of the nanowire which reduces the interface charge states and charged hydroxyl sites.^21,22^ The hysteresis is calculated by taking the corresponding gate voltage value from the center of the subthreshold regime during successive forward and reverse voltage sweeps. This records the dynamics of charge trapping and detrapping in the nanowire channel and the interface. As seen from the graph in Figure 4b, the n- and p-conduction hysteresis reduces from 13 and 28 V, respectively, for back-gated unpassivated state to approximately 2.4 and 6.8 V for the hBN-encapsulated top-gated measurement. Simultaneously, the on-current (*I*_ON_) and on–off ratio (*I*_ON_/*I*_OFF_) levels are enhanced after the passivation of the single nanowire by hBN and with the measurements recorded through the top gate. This is reflected by the modest increase in the n-type on–off ratio and 1 order of magnitude increase in the p-type ratio for the hBN-passivated top-gated measurements. The convention used for calculating I_ON_ and I_OFF_ is explained in detail in the Supporting Information (see Figure S1). The extracted electrical parameters of the hBN-passivated single nanowire-based device with different gating schemes are listed in Table 2.

**Table 1:** SS Values of the hBN-Passivated Single Nanowire-Based Device Using Different Gating Schemes

SS (V/dec)			
carrier type	back gate (without hBN)	back gate (with hBN)	top gate (with hBN)
n	1.82	1.68	0.97
p	1.07	0.78	0.50

**Table 2:** Extracted Parameters from the Transfer Characteristics of the hBN-Passivated Single Nanowire-Based Device at a *V*_DS_ of 0.5 V Using Different Gating Schemes

parameter	carrier type	back gate (without hBN)	back gate (with hBN)	top gate (with hBN)
*I*_ON_ (A)	n	5.8 × 10^–^^9^	2.4 × 10^–^^9^	5.7 × 10^–^^9^
	p	8.4 × 10^–^^9^	1.8 × 10^–^^8^	2.6 × 10^–^^8^
*I*_OFF_ (A)	n	7.8 × 10^–^^16^	7.6 × 10^–^^16^	8.4 × 10^–^^16^
	p	8.2 × 10^–^^16^	1.2 × 10^–^^15^	8.4 × 10^–^^16^
*I*_ON_/*I*_OFF_ ratio	n	∼10^7^	∼10^7^	∼10^7^
	p	∼10^7^	∼10^7^	∼10^8^
hysteresis (V)	n	13	6.2	2.4
	p	28	13.5	6.8

The output characteristics for the single nanowire-based device are shown in Figure S2. The corresponding off-state output characteristics are evaluated by a two-probe measurement for a *V*_DS_ sweep of 0 to 0.5 V keeping the *V*_G_ constant at 0 V (see Figure S2a). Three separate measurements are carried out at different times. The first measurement is before hBN is transferred on the nanowire, the second is after hBN is transferred, and the final measurement is after the top gate is fabricated. For the first two measurements (at such negligible *V*_G_), a near linear regime is exhibited with a considerable increase in the drain current (*I*_D_) after the passivation of the nanowire with hBN. For the final measurement after the top gate is fabricated, it is seen that the *I*_D_ has a significant increase in current with a characteristic showing Schottky type supra linear shape. Since the top gates help in better hBN encapsulation of the single nanowire, better dielectric passivation is expected. This might have led to superior charge carrier transport through the nanowire channel without the influence of the gate voltages. Simultaneously, the on-state output characteristics of the hBN ambipolar device are also measured based on the top-gated transfer characteristics. For the n-type characteristics, *V*_DS_ is varied from 0.25 to 1 V while increasing the *V*_TG_ from 0 to 10 V in steps of 1 V (Figure S2b). For the p-type behavior, similar voltage steps are maintained but in a negative direction (see Figure S2c). A clear Schottky behavior is seen in both types of conduction for high values of *V*_DS_, which provide a fair conclusion to the contact properties based on the ambipolar shape of the transfer characteristics. For the hole conduction, saturation is seen for low *V*_TG_ values. It is also to be noted that in both cases, *I*_D_ increases with an increase in *V*_TG_. This implies that high *V*_TG_ enables more band bending at the Schottky junctions, leading to increased tunneling of carriers. This eventually leads to a higher current flow.

Additionally, to realize the role of the device performance based on the hBN thickness, two hBN-passivated single nanowire-based devices are compared. These two devices exhibit contrasting hBN thicknesses, with one measuring 10 nm and the other measuring 20 nm. The top-gated transfer characteristics of these two devices are obtained and are shown in the Supporting Information Figure S3. For both devices, the *V*_TG_ is swept in a butterfly loop while varying the *V*_DS_ from 0.25 to 1 V in steps of 0.25 V. It is clearly prominent from both the graphs that the devices exhibit ambipolar behavior with a distinctive rise in the I_ON_ levels for both the branches with the increase in *V*_DS_. However, compared to the device with 20 nm hBN as a dielectric, the one with 10 nm shows higher on-currents, lower hysteresis, and better SS. The on–off ratio (*I*_ON_/*I*_OFF_) levels for the 10 nm hBN device are visible for more than one decade compared to the 20 nm hBN device. This is understandable since thinner hBN would provide better passivation and encapsulation to the single nanowire. This will, in turn, lead to the device exhibiting better electrical conduction due to reduced charge scattering. Furthermore, considering the measurements are conducted by top gating through the hBN dielectric, it is expected of the device with the thinner hBN to have a better capacitive coupling. This, in turn, improves the SS of the 10 nm hBN device compared to the 20 nm one. Finally, it is also observed that the hysteresis of the n- and p-branch is much lower for the thinner hBN device hinting at the absence of interface trap states and oxide charges due to the better passivation of the nanowire. In these scenarios, it is clearly understood that the tunability and reconfigurability of the devices are unaltered for either thickness of the hBN. However, the variability in thickness does play a vital role in enhancing the device’s electrical performance. These results serve as a proof of concept that hBN can be employed as a gate dielectric for one-dimensional silicon nanowire-based devices, presented for the first time to the best of our knowledge. The electrostatic coupling can be further enhanced by improving the interface between hBN and the silicon nanowire. The quality, thickness, and uniformity of the hBN can also impact the device’s performance. To achieve this, a well-controlled deposition of hBN using chemical vapor deposition (CVD) or epitaxy can be employed.^23^ Furthermore, nanowires having smooth edges instead of sharp cuts can enable better contact between nanowire surfaces and hBN. Moreover, the properties of hBN can also be tuned, e.g., by doping or defect-induced variations.^24^ This attribute can be exploited to flexibly fabricate devices to attain the desired performance.

**Figure 5:**
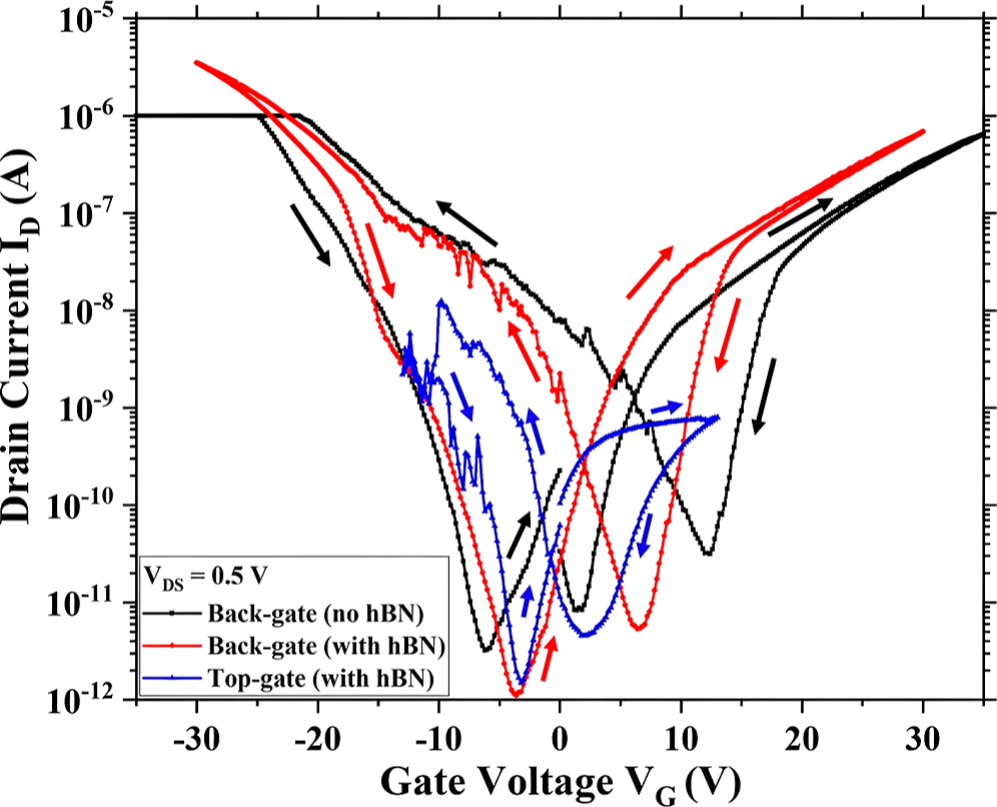
Transfer characteristics of a device consisting of a nanowire array. hBN is used as a dielectric layer. The lengths and widths of the nanowires are 3 μm and 25 nm, respectively. The pitch between the nanowires is 200 nm. The black curve shows a straight line below −20 V due to the compliance set in the measurement tool. For the rest of the transfer characteristics, the compliance was set at a higher drain current value. Similar to Figure 4b, the arrows in the transfer characteristics denote the direction of the curve based on the gate voltage sweep.

To further understand the contact between the nanowires and hBN, the dielectric is also transferred on a nanowire array-based device. The array has 20 nanowires with a pitch of 200 nm. The transfer characteristics are listed in Figure 5. The three gating schemes are used similarly to the single nanowire-based device. With back gating, the device with the hBN flake showed an improvement in on- and off-currents and, hence, in the on–off ratio by nearly 2 orders of magnitude (Table 4). This means that passivation by hBN of the 20 nanowires helps to improve these parameters. Similarly from Figure 5, it is seen that the shift of the transfer curve minima is largely reduced. Subsequently, the hysteresis of the device reduces from its unpassivated state to hBN-passivated top gate state (see Table 4) exactly analogous to how it was observed for the single nanowire-based device. However, it is also to be noted that compared to the single nanowire-based device, the nanowire array-based device has higher off-currents (almost 4 orders of magnitude). The reason for this can be that the minimum current *I*_OFF_ is dependent on the *V*_DS_ value, with *V*_G_ being negligible. Therefore at room temperature, with a considerable *V*_DS_ and negligible *V*_G_, an adequate amount of thermally activated charge carriers can pass through the drain-source contacts. Nonetheless, for a single nanowire, this current value is low. However, it is understandable that the array-based device consists of 20 single nanowires in parallel. Each of these nanowires contributes charge carriers thermionically in the off-state, thus increasing the off-current. Furthermore, the device maintained the off-current when the transfer characteristics were obtained with top gating, but the on-current was reduced. This is attributed to the weak interface between nanowire arrays and hBN (Supporting Information Figure S4). Since nanowires have a pitch of 200 nm, hBN is seen to cover only the top part of the nanowires and not the sidewalls. With an increasing pitch of the nanowires, the hBN coverage of the nanowires can be expected to improve and vice versa. It is expected that for nanowire arrays with a larger distance between the nanowires, a thinner hBN would bend in the gap. This would provide passivation and encapsulation from all three sides of the nanowire. However, with a low pitch of 200 nm, the hBN only sits on the top of the nanowire and does not encapsulate it like in the case of the single nanowire-based device. Thus, although back-gated characteristics improved due to the added effect of the multiple nanowires, the top gate result degraded due to weak electrostatic coupling. This is reflected in the SS values of these measurements (Table 3). The SS values improved after hBN transfer with back gate measurements. Although both these measurements provide capacitive gate coupling through the same back gate, the measurement after hBN transfer has a better SS value due to the surface passivation of the nanowires by hBN. With hBN reducing the interface state density of the top surface of the nanowire, the SS value is strongly influenced.^25^ However, the SS of the n-branch degraded with the top gate measurements compared to its back gate due to lower gate coupling through the hBN on the multiple nanowires.

**Table 3:** SS Values of hBN-Passivated Nanowire Array-Based Devices Using Different Gating Schemes

SS (V/dec)			
carrier type	back-gate (without hBN)	back-gate (with hBN)	top-gate (with hBN)
n	2.56	1.76	3.05
p	5	3.06	1.5

**Table 4:** Extracted Parameters from the Transfer Characteristics of the hBN-Passivated Nanowire Array-Based Device at a *V*_DS_ of 0.5 V Using Different Gating Schemes

parameter	carrier type	back gate (without hBN)	back gate (with hBN)	top gate (with hBN)
*I*_ON_ (A)	n	6.4 × 10^–^^7^	6.8 × 10^–^^7^	8.0 × 10^–^^10^
	p	7.3 × 10^–^^8^	2.7 × 10^–^^7^	8.2 × 10^–^^9^
*I*_OFF_ (A)	n	3.2 × 10^–^^11^	5.3 × 10^–^^12^	4.5 × 10^–^^12^
	p	3.2 × 10^–^^11^	5.3 × 10^–^^12^	4.5 × 10^–^^12^
*I*_ON_/*I*_OFF_ ratio	n	∼10^4^	∼10^5^	∼10^2^
	p	∼10^3^	∼10^5^	∼10^3^
hysteresis (V)	n	8	7.6	6.9
	p	16	11	4.2

Furthermore, a gate leakage current analysis is performed during each measurement step to prevent the flow of high current through the dielectric layer during continuous voltage scans. The repeated flow of high currents can eventually lead to dielectric breakdown and degradation of the hBN-passivated nanowire-based devices. The measured leakage current analysis for hBN-passivated single nanowire- and nanowire array-based devices with different gating schemes are shown in Supporting Information Figure S5. For both the single nanowire- and nanowire array-based devices, the leakage current is measured by sweeping the gate voltage from a low potential to a high potential. Repeated voltage scans that produce currents above 10 pA can be fatal for the dielectric and the device. Therefore, compliance of 10 pA was set in the measurement setup prior to the analysis. As seen from Figure S5, the gate leakage current I_G_ (denoted by the red curve) is always under the set limit of 10 pA and showed minimal leakage through the gate dielectric. In the scope of this work, a total of 18 nanowire devices are fabricated. Out of 12 are single nanowires and 6 are nanowire array devices. Exfoliated hBN is successfully transferred to 12 of the devices, while the top gate is fabricated on 5 of them. Out of the five top-gated single nanowire-based devices, three are nominally identical with hBN thickness varying between 10 and 15 nm. The other two devices had significantly thicker hBN gate dielectric, which led to weaker gate coupling and considerably worse device characteristics (as explained in the earlier section). On the other hand, the three devices with thinner hBN consistently exhibited similar device performances. The electrical parameters of these nominally identical three devices are extracted to derive the statistical analysis including the mean values and standard deviations (SD). This is shown in Supporting Information Table S1. Figure S6 presents the extracted electrical parameters of the three devices along with their resulting mean and SD values. The SD values in Figure S6a–c are relatively higher than their mean values. This is because the currents extracted have deviations of a couple of orders of magnitude owing to the device-to-device variability. Since the nanowires are fabricated by a top-down approach, these devices are robust and stable. hBN also helps to protect the devices from the outside environment. Therefore, over an extended period of time, the devices maintained consistent functionality and performance. The fabrication of these 12 nanowire devices with hBN is also carried out over different time spans. Their similar device behavior implied the reproducibility of the devices and the reliability of the fabrication process that is employed.

## Experimental Section

**Figure 6:**
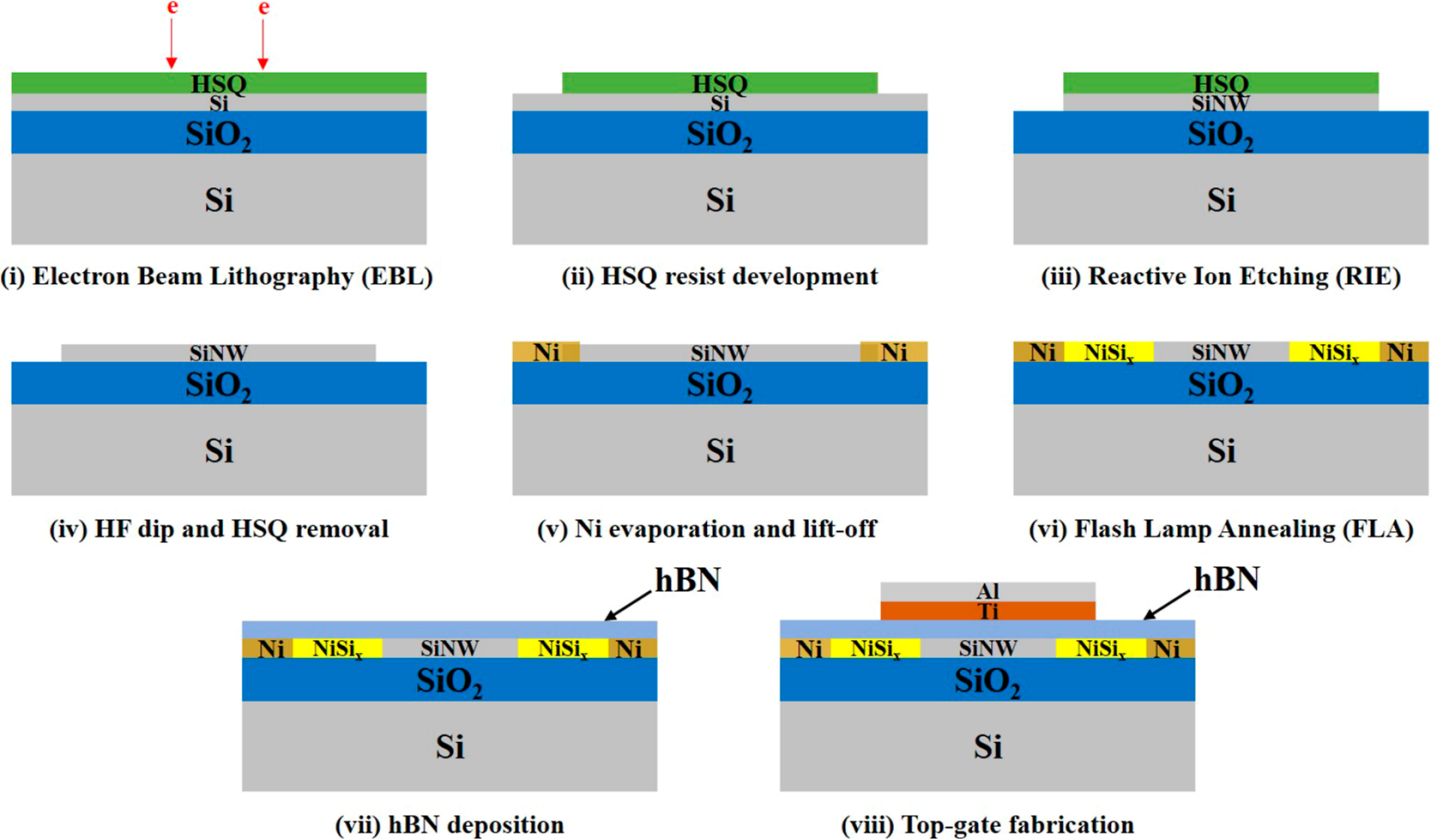
A schematic representation of the top-down fabrication process flow of the novel mixed-dimensional RFET device. (i) Starting with SOI substrate, a negative resist HSQ is spin-coated for patterning with EBL, (ii) development of HSQ resist creates the HSQ patterns in the shape of the nanowire, (iii) HSQ patterns are then transferred to the top 20 nm device layer using anisotropic reactive ion etching, (iv) HF dip is carried out to remove the top HSQ later, (v) source and drain Ni contact pads are fabricated by EBL, metal deposition, and lift-off technique, (vi) FLA creates the NiSi_2_ Schottky junction inside the nanowire, (vii) exfoliated hBN is transferred onto the device by dry-stamping technique, and (viii) top gates are fabricated on top of the hBN layer by EBL, metal deposition, and lift-off.

The substrate material used for the fabrication of a mixed-dimensional RFET is a 1 × 1 cm^2^ silicon-on-insulator (SOI) substrate. It consists of layers of silicon-insulator-silicon stacked on top of each other. The device or active top layer of the substrate consists of 20 nm intrinsic silicon, followed by a 102 nm thick buried oxide (BOx) layer and a 775 μm p-doped Si carrier wafer. In this work, the fabrication of nanowires is based on the top-down approach, which allows the large-scale integration of devices. The top-down approach involves the use of a substrate material, which is subjected to successive subtractive procedures to ultimately achieve a nanostructure. The detailed process of silicon nanowire fabrication is described in our previous work^18^ and schematically shown in Figure 6. At first, the SOI substrate is cleaned thoroughly in various chemicals, including Piranha solution, acetone, isopropanol (IPA), and deionized (DI) water. Then, a 2% hydrogen silsesquioxane (HSQ) (XR-1541 from DuPont) negative-tone resist is spin-coated on the substrate at 2000 rpm for 30 s, creating a 40 nm thick HSQ layer. The nanowires are exposed using a RAITH e-LINE PLUS electron beam lithography (EBL) system at an acceleration voltage of 10 kV, a base dose of 1000 μC cm^–2^, an aperture with the size of 30 μm, and a beam area step size of 2 nm. The exposure parameters are optimized according to the nanowire dimensions and were used throughout this work for patterning the appropriate nanostructures. After the exposure, the substrate is subjected to a resist development, which removes the unexposed resist.^26^ A SENTECH inductively coupled plasma reactive ion etching (ICP-RIE) Si 500 system is used to anisotropically transfer the HSQ pattern to the active device layer. The fabrication of source and drain Ni contacts at both ends of the nanowire includes a similar process of EBL patterning, metal deposition using a UHV e-beam evaporation system from BESTEC, and a lift-off process. Flash lamp annealing (FLA) is performed on the substrate after the Ni contact deposition to achieve diffusion of Ni into the nanowire to form the NiSi_2_ phase.^18^ Ni silicidation in the nanowire creates a heterostructure of NiSi_2_–Si–NiSi_2_ forming two Schottky junctions on either side. A prior study was conducted to develop a Ni silicidation process of silicon nanowires by rapid thermal annealing (RTA).^27^ This study was further continued to compare the results with FLA.^18^ The optimization of the FLA parameters with different energy densities, flash pulse durations, and various inert gas conditions was performed to achieve homogeneous Ni silicide formation in the nanowire. The optimum FLA treatment is obtained for the energy density of about 89 J cm^–2^ for 6 ms in continuous nitrogen flow.

**Figure 7:**
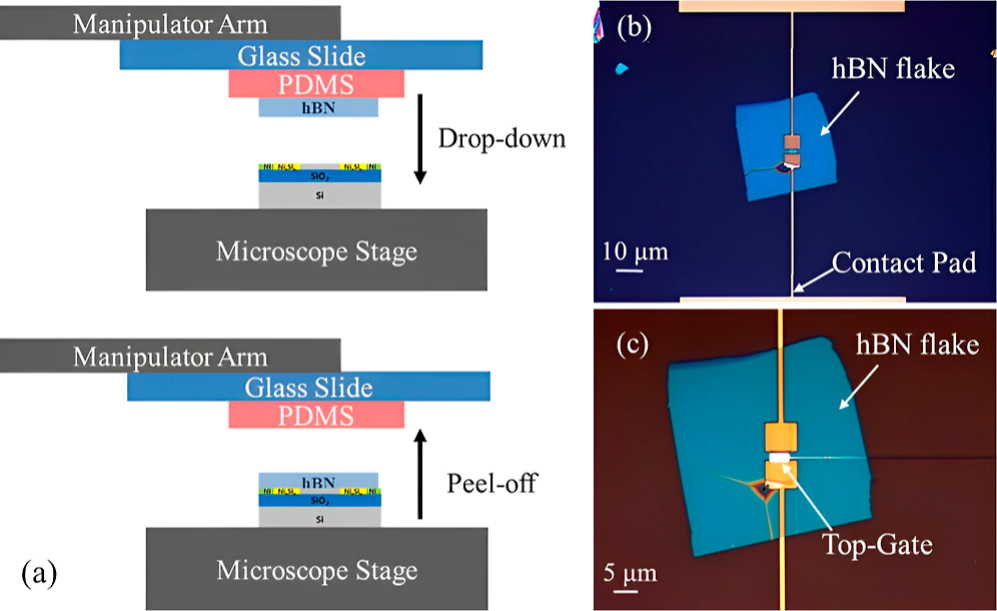
(a) Transfer of hBN flake indicating the steps of drop-down and peel-off process onto the silicon nanowire-based devices using the dry viscoelastic stamping transfer technique. (b) Visible-light micrograph image of a transferred hBN flake on a single silicon nanowire-based device showing the architecture of contact pads and connection lines. (c) Visible-light micrograph image of a mixed-dimensional RFET device with a top-gate present on the hBN layer.

The hBN flakes are prepared using the mechanical exfoliation technique^28^ on a polydimethylsiloxane (PDMS) substrate (approximately 1 × 1 cm^2^) employing commercial scotch tape. Suitable hBN flakes are then identified with the help of an optical microscope based on their thickness, lateral dimensions, and uniformity. The hBN flakes are transferred onto the nanowire using the dry-stamping technique^29^ by aligning the desired flake onto the nanowire with the help of a micromanipulator setup that is coupled to an optical microscope. The schematic of the hBN transfer process and optical micrographs of the mixed-dimensional RFET devices are shown in Figure 7. With favorable hBN flakes on the silicon nanowires, single top gates are placed based on EBL patterning, metal deposition, and lift-off processes. A stack of titanium (Ti) and aluminum (Al) is typically used as the top gate.

Bright-field transmission electron microscopy (TEM) images are recorded using an image-C_s_-corrected TITAN 80-300 microscope (FEI) operated at an accelerating voltage of 300 kV. With a TALOS F200X microscope equipped with an X-FEG electron source and a Super-X EDX detector system (FEI), high-angle annular dark-field scanning TEM (HAADF-STEM) imaging and spectrum imaging analysis based on energy-dispersive X-ray spectroscopy (EDXS) are performed at 200 kV. Before (S)TEM analysis, the specimen mounted in a high-visibility low-background holder is placed for 8 s into a FISCHIONE 1020 plasma cleaner to eliminate any potential contamination. Cross-sectional TEM specimens of the mixed-dimensional RFET devices are prepared by in situ lift-out using a Helios 5 CX-focused ion beam (FIB) device (Thermo Fisher). To protect the sample surface, a carbon cap layer is deposited beginning with electron-beam-assisted and subsequently followed by Ga-FIB-assisted precursor decomposition. Afterward, the TEM lamella is prepared using a 30 keV Ga-FIB with adapted currents. Its transfer to a 3-post copper lift-out grid (Omniprobe) is done with an EasyLift EX nanomanipulator (Thermo Fisher). To minimize sidewall damage, Ga ions with only 5 keV energy are used for the final thinning of the TEM lamella to electron transparency. Tapping mode AFM analysis is carried out using a NANOFRAZOR SCHOLAR tool from HEIDELBERG INSTRUMENTS. For the electrical transfer characteristics measurements, a semiautomated SÜSS MICROTECH probe system PA200 connected with a 4200-SCS KEITHLEY INSTRUMENTS characterization system is used.

## Summary

A novel hybrid mixed-dimensional RFET concept is demonstrated by introducing 2D hBN in nanowire-based electronics. The fabricated devices exhibit an improvement in SS values, a reduction in hysteresis, and an enhancement of the p and n on-currents. To conclude, hBN can be used as an effective dielectric and passivating layer for one-dimensional nanowire devices. Since the properties of the hBN can be tuned, versatile devices can be fabricated using this robust material. However, optimization of the device design and control over the hBN thickness is required for superior device performance. This first demonstration of hBN incorporation in nanowire-based devices potentially opens up a new paradigm in semiconductor electronics. Furthermore, system-level integration of these devices can be achieved using CVD or epitaxy-based deposition of hBN.

## Supporting Information Available

Information on the convention for calculation of on- and off-currents, on-state and off-state output characteristics, transfer characteristics based on different hBN thicknesses RFET devices, additional TEM and EDXS image of hBN-passivated silicon nanowire array-based device, and leakage current analysis (PDF)
