# Supplementary material for: Novel Mixed-Dimensional hBN-Passivated Silicon Nanowire Reconfigurable Field Effect Transistors: Fabrication and Characterization
Source: ACS Appl Mater Interfaces. 2023 Aug 22;15(34):40709–18. doi: 10.1021/acsami.3c04808 (PMC10472425; doi:10.1021/acsami.3c04808)
Supplement: Supplementary file 1 — am3c04808_si_001.pdf [file am3c04808_si_001.pdf]

Supporting Information
Novel Mixed-Dimensional hBN-Passivated Silicon Nanowire Reconfigurable Field Effect Transistors: Fabrication and Characterization

Sayantan Ghosh,[*,†,‡] Muhammad Bilal Khan ,[†] Phanish Chava,[†,‡] Kenji Watanabe,[¶] Takashi Taniguchi,[¶] Slawomir Prucnal,[†] René Hübner,[†] Thomas Mikolajick,[§,‡,∥] Artur Erbe,[†,‡,∥] and Yordan M. Georgiev[*,†,‡,⊥]

†*Institute of Ion Beam Physics and Materials Research, Helmholtz-Zentrum Dresden-Rossendorf (HZDR), Bautzner Landstraße 400, Dresden, 01328, Germany*

‡*Technische Universität Dresden, Dresden, 01069, Germany*

¶*National Institute for Materials Science, 1-1 Namiki, Tsukuba 305-0044, Japan*

§*Namlab gGmbH, Nöthnitzer Strasse 64, Dresden, 01187, Germany*

∥*Technische Universität Dresden, Center for Advancing Electronics Dresden (CfAED), Dresden, 01069, Germany*

⊥*Institute of Electronics at the Bulgarian Academy of Sciences, 72, Tsarigradsko chaussee blvd., Sofia 1784, Bulgaria*

E-mail: s.ghosh@hzdr.de; y.georgiev@hzdr.de

In the transfer characteristics, the on-current (I_{ON}) is defined as the saturation current in the active mode of operation. The on-current for either the p-type or the n-type conductance is determined from the point where the transfer curve goes into saturation. The off-current

(I_{OFF}) is the point where the device switches off. However, there is a presence of leakage current between the source and drain terminals when the gate voltage is below the threshold voltage (in the subthreshold regime). In the case of the devices measured, there is a shift in the transfer characteristics. Hence, this shift is considered for calculating the on and the off-currents. Depending on the voltage side the shift is in (either positive or negative V_{TG}), the peak on-current for that specific branch is calculated at the maximum voltage which can be reached. For the other branch, the voltage shift value is subtracted from its maximum voltage (opposite polarity) to determine its peak on-current. An example of the on and off-current calculation is shown in figure S1.

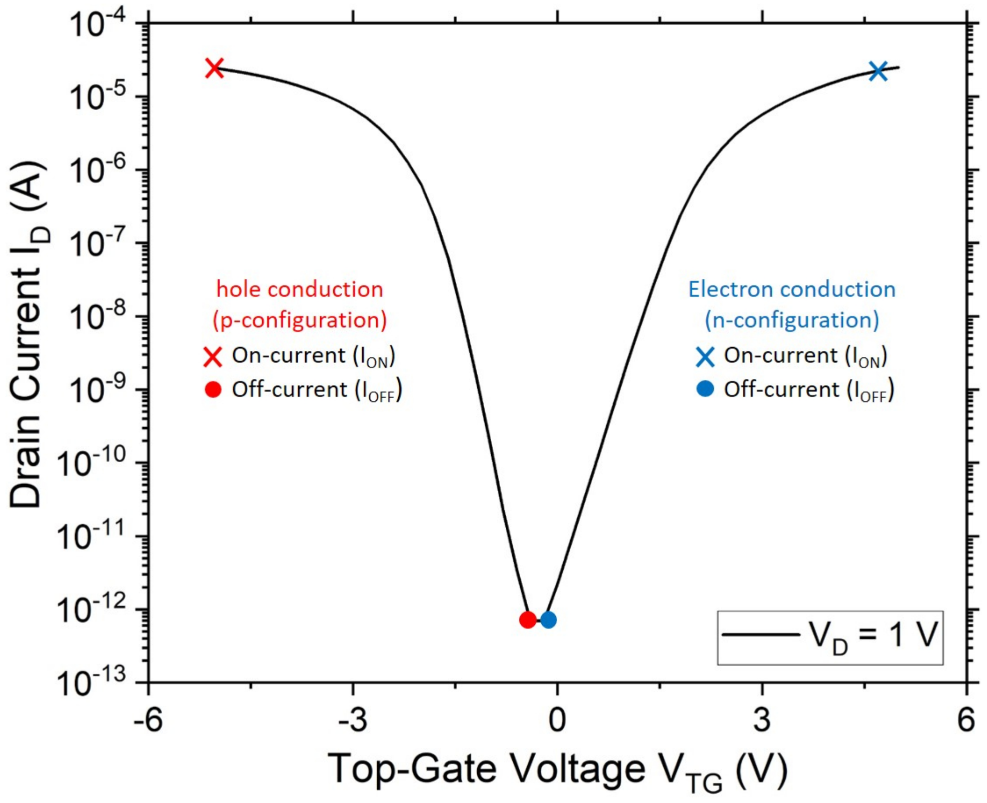

Figure S1: Convention used for the calculation of on and off-current from transfer characteristics of hBN RFET devices

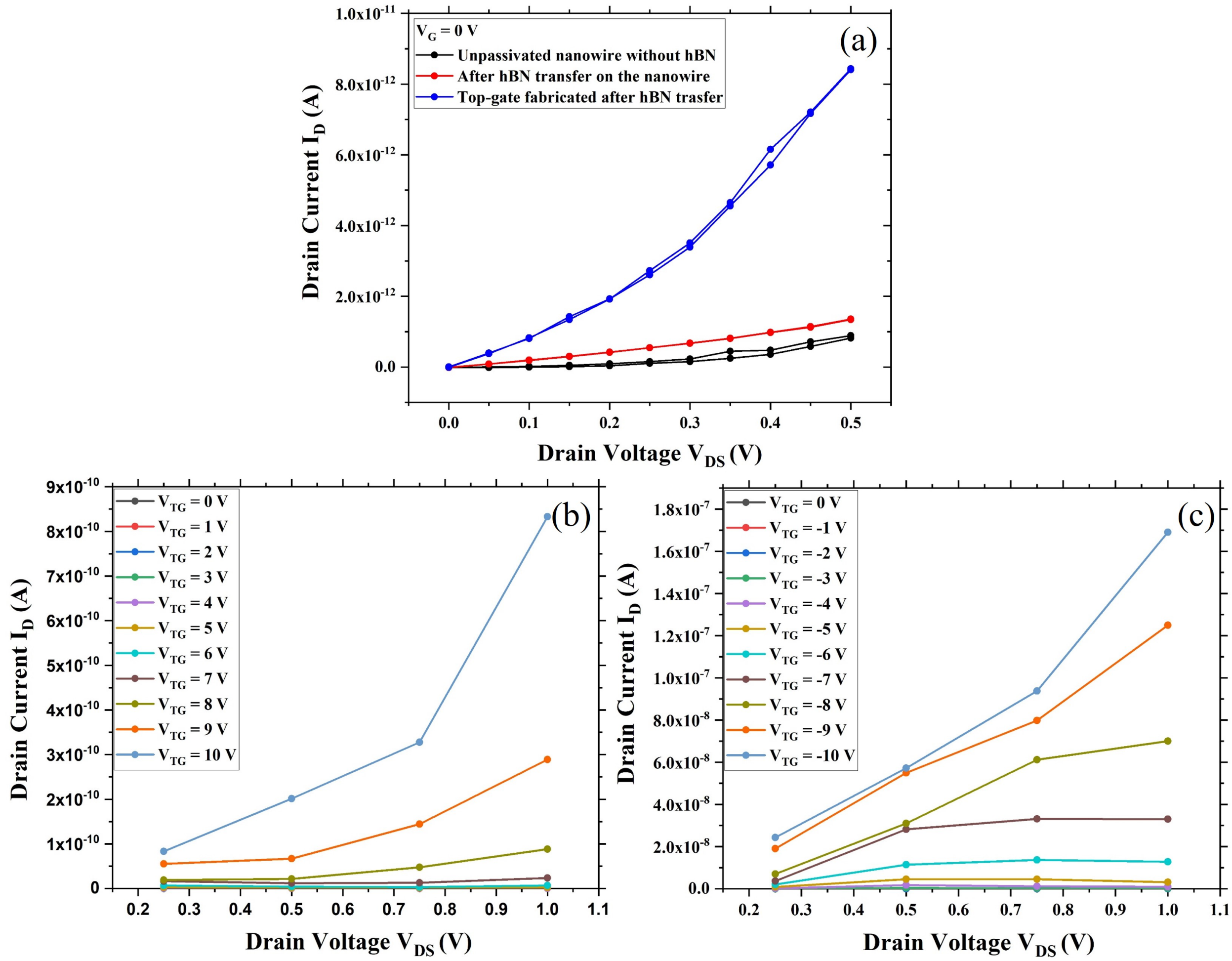

Figure S2: Output characteristics (I_D-V_{DS}) of a single silicon nanowire-based device comprising hBN flake as the dielectric layer. The length and width of the nanowire are 3 µm and 25 nm, respectively (a) Off-state output characteristics of the device comprising measurements taken before and after hBN is transferred on the single nanowire and after the top gate is fabricated (b) On-state n-type output characteristics of an hBN single nanowire-based device, indicating a Schottky-type behaviour. The top gate voltages are varied from 0 to 10 V in steps of 1 V (c) On-state p-type output characteristics of the same hBN single nanowire-based device, indicating a Schottky-type behaviour. The top gate voltages are varied from 0 to -10 V in steps of -1 V.

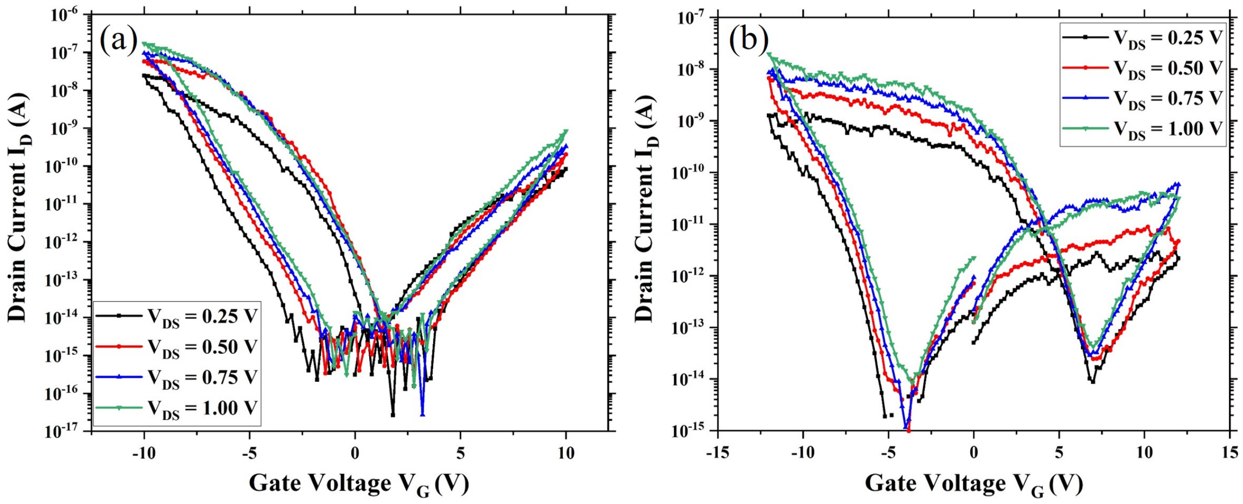

Figure S3: Comparison of transfer characteristics (I_D-V_G) with different source-drain voltages (V_{DS}) for a single nanowire-based device with (a) 10 nm thick hBN flake as the gate dielectric and (b) 20 nm thick hBN flake as the gate dielectric. The V_{DS} value is varied in each measurement step from 0.25 to 1 V in steps of 0.25 V.

A lower magnification scanning TEM (STEM) image is captured for the nanowire array-based device and is shown in figure S4 (a). The hierarchy of the device starts with the p-type carrier Si wafer with a buried SiO_2 layer on top of it. The silicon nanowires are on top of the buried oxide layer. 4 out of 20 nanowires in the array are imaged. Figure S4 shows that the hBN flake positions itself in a planar way on top of the nanowires. No curvature of the hBN is seen, unlike the single nanowire-based device. The thickness of the hBN flake is approximately 20 nm. Based on the structure of the hBN flake, the top gates consisting of Ti and Al are also positioned in a planar fashion. Furthermore, the STEM image confirms the distance between the nanowires to be 200 nm. Figure S4 (b) shows the superimposed EDXS-based element distribution maps for the field of view depicted in Figure S4 (a). The region with B and N confirms the presence of the hBN layer with a dual layer of Ti and Al on top of it. The hBN layer sits on top of the nanowires without conformally encapsulating them. This results in a weak interface between hBN and the Si nanowires leading to lower electrostatic coupling and degradation in device performance compared to

the single nanowire-based device.

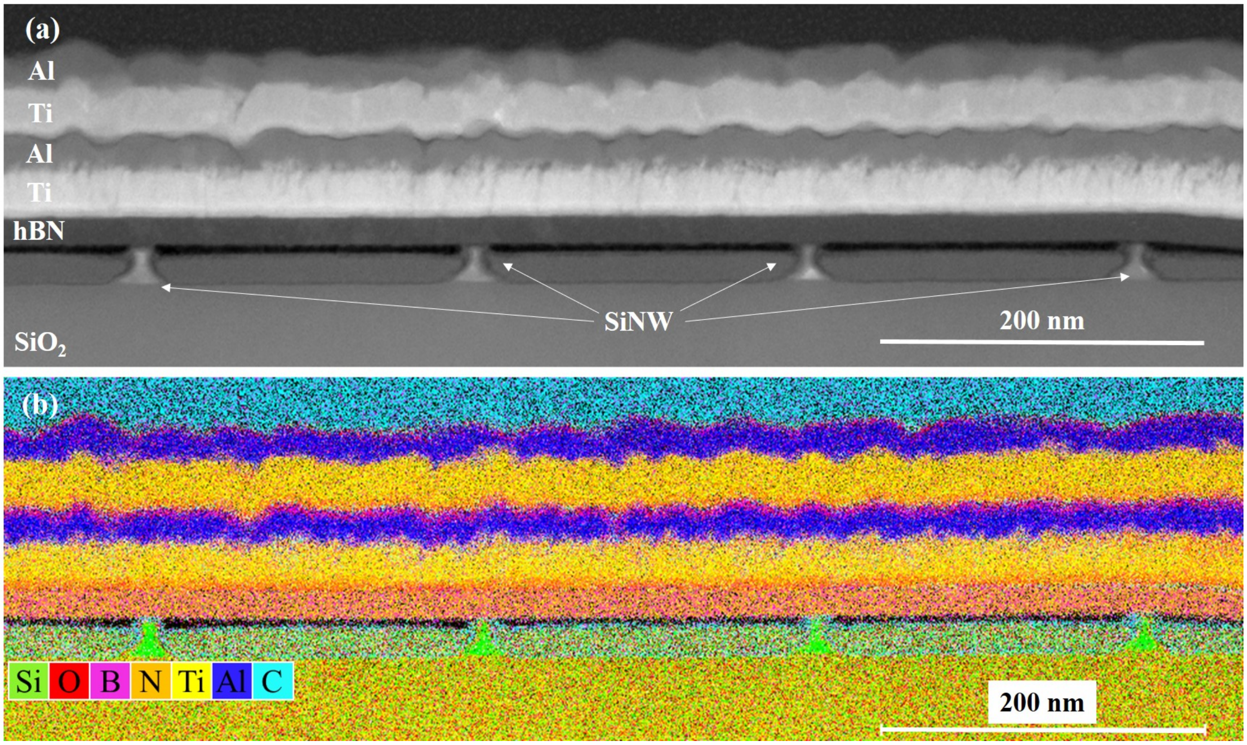

Figure S4: (a) HAADF-STEM micrograph of a sectioned nanowire array-based device showing the Si carrier wafer, a buried oxide layer, 4 out of 20 nanowire channels, the hBN dielectric layer, and the dual top gate structure. (b) Corresponding superimposed EDXS-based element distribution maps of the hBN-nanowire array-based device.

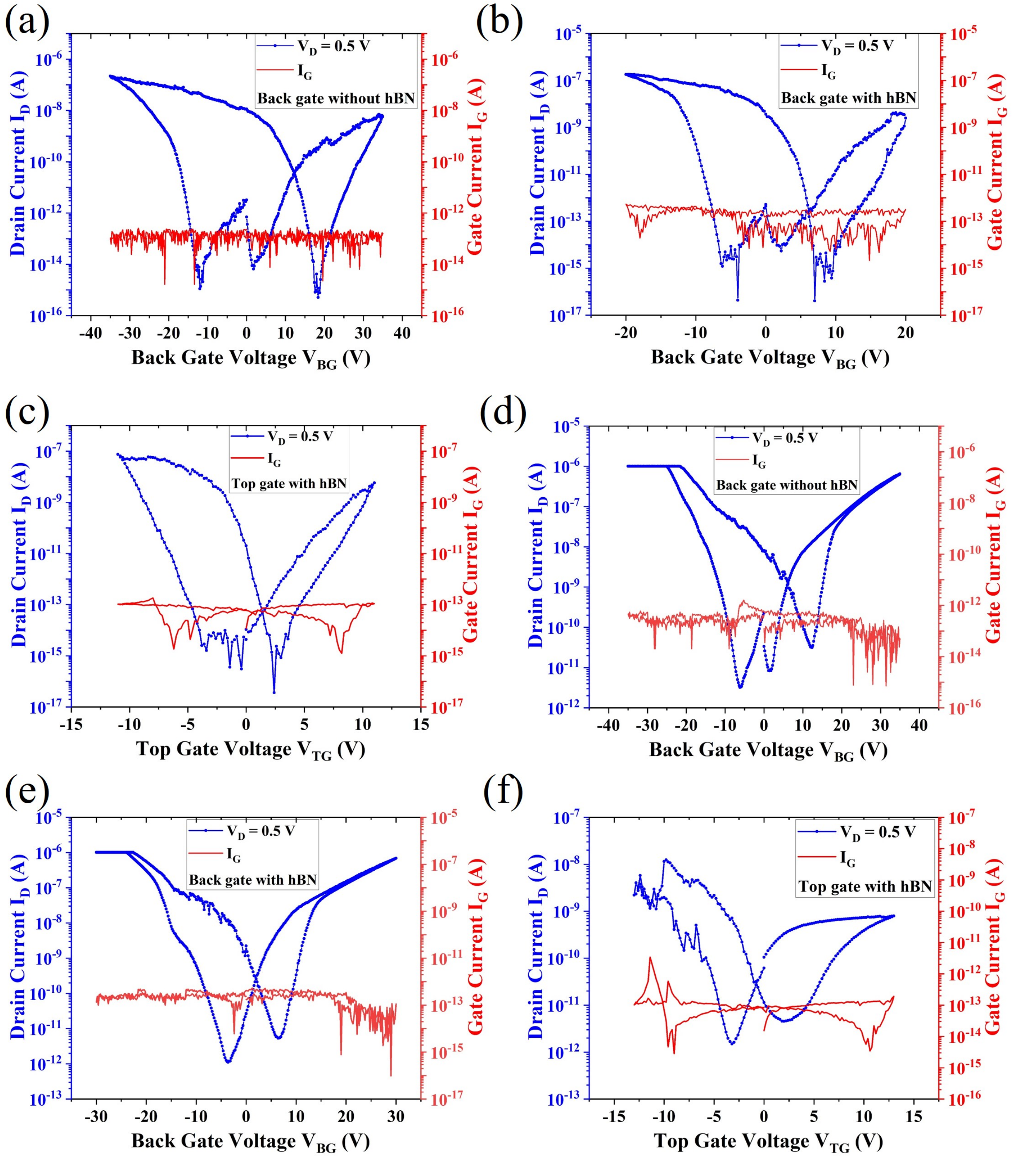

Figure S5: Leakage current analysis during every transfer characteristics measurement for a single nanowire-based device in the scenario of (a) Back gate measurements without hBN (b) Back gate measurements after hBN is transferred and (c) Top gate measurement with hBN as the primary gate dielectric. Similarly, leakage analysis is also carried out for a nanowire array-based device in the scenario of (d) Back gate measurements without hBN (e) Back gate measurements after hBN is transferred and (f) Top gate measurement with hBN as the primary gate dielectric. The blue curve denotes the transfer characteristics axis and the red curve denotes the gate leakage current axis.

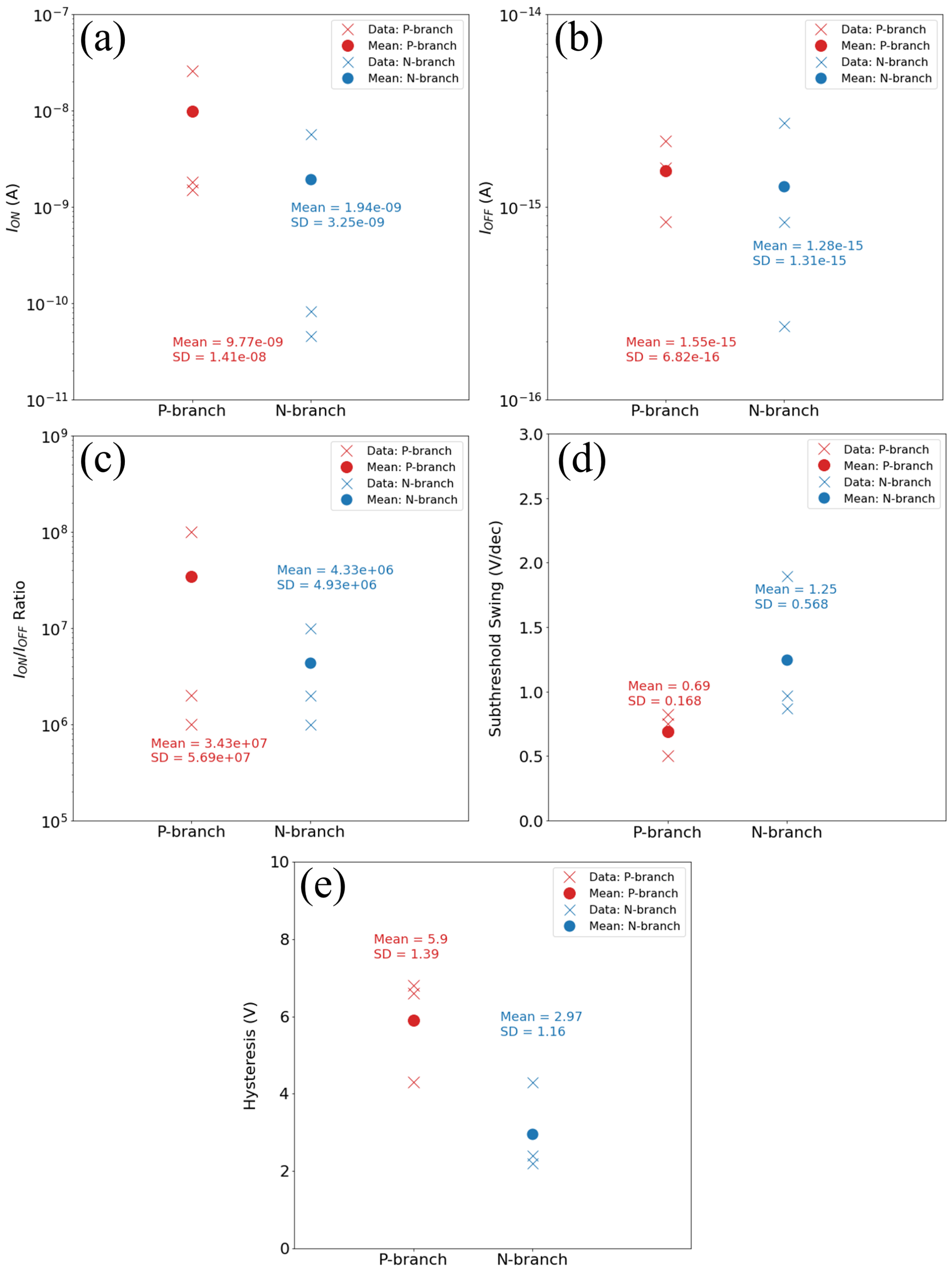

Figure S6: The extracted electrical parameters: (a) On-current I_{ON} (b) Off-current I_{OFF} (c) On-current to off-current (I_{ON}/I_{OFF}) ratio (d) Subthreshold swing and (e) Hysteresis along with their mean and standard deviation values. Each plot shows values for both the p- and n-branches of the 3 nominally identical hBN-passivated single-nanowire based top-gated devices.

Table S1: The mean and the standard deviation (SD) of 3 nominally identical hBN-passivated single-nanowire based top-gated devices.

Parameter	Mean		Standard Deviation (SD)	
	n	p	n	p
I_{ON} (A)	1.94 x 10^{-9}	9.77 x 10^{-9}	3.25 x 10^{-9}	1.41 x 10^{-8}
I_{OFF} (A)	1.28 x 10^{-15}	1.55 x 10^{-15}	1.31 x 10^{-15}	6.82 x 10^{-16}
I_{ON}/I_{OFF} Ratio	4.33 x 10^{6}	3.43 x 10^{7}	4.93 x 10^{6}	5.69 x 10^{7}
Subthreshold Swing (V/dec)	1.25	0.69	0.568	0.168
Hysteresis (V)	2.97	5.90	1.16	1.39
